# Design of Ethylene-Vinyl Acetate Copolymer Fiber with Two-Way Shape Memory Effect

**DOI:** 10.3390/polym11101599

**Published:** 2019-09-30

**Authors:** Xiaoming Qi, Wentong Yang, Laiming Yu, Wenjun Wang, Haohao Lu, Yanglong Wu, Shanwen Zhu, Yaofeng Zhu, Xiangdong Liu, Yubing Dong, Yaqin Fu

**Affiliations:** Key Laboratory of Advanced Textile Materials and Manufacturing Technology Ministry of Education, Zhejiang Sci-Tech University, Hangzhou 310018, China; xmqi19931127@163.com (X.Q.); 819177316ywt@163.com (W.Y.); kyokylin@126.com (L.Y.); wangwj310@163.com (W.W.); mrluhaohao@163.com (H.L.); mrwyl0818@163.com (Y.W.); zswae86@163.com (S.Z.); yfzhu@zstu.edu.cn (Y.Z.); liuxd@zstu.edu.cn (X.L.)

**Keywords:** shape memory polymer fiber, two-way shape memory effect, melt spinning, UV curing

## Abstract

One-dimensional shape memory polymer fibers (SMPFs) have obvious advantages in mechanical properties, dispersion properties, and weavability. In this work, a method for fabricating semi-crystallization ethylene-vinyl acetate copolymer (EVA) fiber with two-way shape memory effect by melt spinning and ultraviolet (UV) curing was developed. Here, the effect of crosslink density on its performance was systematically analyzed by gel fraction measurement, tensile tests, DSC, and TMA analysis. The results showed that the crosslink density and shape memory properties of EVA fiber could be facilely adjusted by controlling UV curing time. The resulting EVA fiber with cylindrical structure had a diameter of 261.86 ± 13.07 μm, and its mechanical strength and elongation at break were 64.46 MPa and 114.33%, respectively. The critical impact of the crosslink density and applied constant stress on the two-way shape memory effect were analyzed. Moreover, the single EVA fiber could lift more than 143 times its own weight and achieve 9% reversible actuation strain. The reversible actuation capability was significantly enhanced by a simple winding design of the single EVA fiber, which provided great potential applications in smart textiles, flexible actuators, and artificial muscles.

## 1. Introduction

Smart materials as an emerging key technology, will provide unique capabilities for new products. The special feature of smart materials is active “smart” response rather than sensing, such as self-sensing, self-healing, self-actuating, self-diagnostic, and shape changing [1]. As an important branch of smart materials, shape memory polymers (SMPs) can memorize temporary shape and return to their initial shape when exposed to external stimuli (such as heat, light, electricity, magnetic field, solvent, and pH) [2,3,4,5,6,7,8,9,10,11]. Furthermore, SMPs have extraordinary advantages due to its light weight, low cost, flexibility, large deformation, and easy manufacture process compared to shape memory alloys and shape memory ceramics. These unique properties have potential in diverse applications, ranging from aerospace [5], sensors [12,13], and actuators [14,15] to smart textiles [16,17], self-healing [18,19], and artificial muscles [20,21].

Over the past few decades, a variety of structural SMPs have emerged, including shape memory films [22], shape memory foams [23,24,25], and shape memory polymer fibers (SMPFs) [16,26,27]. Although shape memory films and foams have their own superiority and applications, the one-dimensional orientation of SMPFs with excellent mechanical properties, greater recovery stress, and faster response speed greatly enhances the functionality of SMPs [28]. Li et al. proposed that SMPFs have good mechanical properties and dispersibility, and it provided excellent self-healing ability for the intelligent composites [29,30]. When the composites were damaged, the driving force for self-healing was supplied by the SMPFs. The cold-drawing SMPFs restore the shrinkage stress when the composites were heated above the glass transition temperature of the SMPFs. Moreover, SMPFs can be woven into smart comfort-adjusting textile and actively adjust their porosity in response to changes in the external environment [31,32].

SMPFs are mainly fabricated from thermoplastic SMPs, especially polyurethane (PU). SMPFs based on PU with micro-phase-separated structure, in which the hard segment as the physical crosslink and the soft segment as the transition phase memory temporary shape. The response temperature and mechanical properties of the PU fiber can be adjusted by varying the ratio of hard and soft segments [33]. Recently, two-way SMPs (2W-SMPs) based on semi-crystalline polymers have achieved great progress. 2W-SMPs can exhibit a two-way shape memory effect (2W-SME) without external programming [34,35,36]. This feature is expected to make 2W-SMPs an ideal candidate for actuators, artificial muscles, and self-locomotion robots. Combined with intelligent material characteristics and fiber processing techniques, the researchers reported variety of smart twisted fiber with reversible actuation [37,38]. However, the production of large-scale twisted fiber-based artificial muscles in an efficient manner still presents challenges. Therefore, the efficient preparation of intelligent two-way shape memory fibers based on semi-crystalline polymers is one of the research hotspots.

Considering the molecular structure of 2W-SMPs, the premise of fiber with 2W-SME is that the fibers must have crosslinked network. Commercial semi-crystalline polymers such as polyethylene, polypropylene, poly(ε-caprolactone), and ethylene-vinyl acetate copolymer (EVA) have been widely used to design 2W-SMPs due to their low cost and ease of processing [39,40,41,42,43,44]. Our previous research indicated that EVA with crystallization and crosslinked structure exhibited melting induced contraction (MIC) and crystallization induced elongation (CIE) under constant stress and stress-free conditions [42,45]. In general, commercial semi-crystalline EVA can be easily processed into fibers. Therefore, the crosslinked network structure is important to manufacturing EVA fiber with 2W-SME. At present, most researchers fabricated the crosslinked EVA film by using a thermally induced free radical reaction. Upon heating above the thermally induced free radical temperature, the macroscopic shrinkage and plastic flow of EVA fiber would occur due to melt transition. Therefore, it is critical to crosslink EVA fiber at room temperature to avoid the structure damages. In the last five years, SMPFs with nano- or micro-scale fibrous structures were obtained by electrospinning [46,47,48,49], the crosslinked network has been obtained though free radical reaction initiated by ultraviolet (UV). The mild temperature (lower than Tm) of the UV curing avoids losing its fibrous structure while obtaining a crosslinked network. Compared to electrospinning fiber membranes, the single fibers prepared by melt spinning can be assembled in an orderly manner to form a more diverse variety of yarn, braid, and fabric, respectively.

In this study, EVA fiber was first prepared by efficient and convenient melt spinning, benzophenone and triallyl isocyanurate were introduced to EVA fiber as photoinitiator and multi-functional crosslinking agent, respectively. A series of EVA fibers with different crosslink density were prepared by controlling the UV curing time. The effect of the crosslink density on the mechanical, thermal, and shape memory properties of EVA fiber was discussed. In addition, the impact of the crosslink density and the constant stress for 2W-SME were investigated. Moreover, the EVA fiber could lift more than 143 times its own weight and achieve 9% reversible actuation strain. The EVA fiber may have great potential in smart textiles, flexible actuators, and artificial muscles.

## 2. Materials and Methods

### 2.1. Materials

EVA with a vinyl acetate content of 18 wt% and melt index 2.5 g/min (Elvax460, DuPont), benzophenone (BP) (99%) and triallyl isocyanurate (TAIC) (contains 500 ppm BHT stabilizer) were purchased from Aladdin Reagent, Shanghai, China. Xylene (99%) was purchased from commercial (Hangzhou Gaojing Fine Chemical Co., Ltd., Hangzhou, China). All the chemicals were used as received.

### 2.2. Fabrication of EVA Fiber

**Figure 1:**
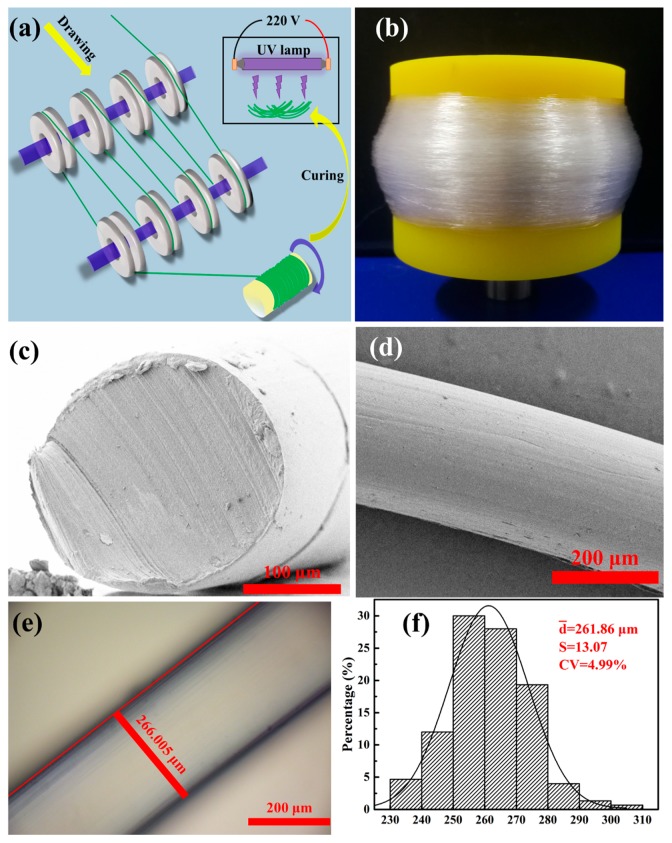
(**a**) Preparation schematic of drawing and ultraviolet (UV) curing; (**b**) digital photograph of ethylene-vinyl acetate (EVA) fiber before UV curing; (**c**,**d**) the SEM images of EVA fiber after UV curing; (**e**,**f**) the optical micrograph and diameter distribution of EVA fiber after UV curing.

EVA, BP, and TAIC were pre-mixed at a mass ratio of 98:1:1, and then, the mixture was melt-extruded using a micro twin-screw extruder (SJSZ-10A, Wuhan Ruiming, Wuhan, China). The temperatures of the extruder heating zone were set to 130 °C, 135 °C, 145 °C, and 150 °C, the screw main engine speed was 40–50 rpm, the feed rod speed was 30–40 rpm. The EVA fiber was drawn and collected by a speed-regulating rotor, which drafting speed at 15–20 m/min. The extrusion speed was 1–2 m/min and extrusion die to collection roller distance was 100 cm. The EVA fibers were separated from the collection roller and cured using a UV lamp with the main wavelength of 365 nm (800 µw/cm^2^), and schematic of the fabrication process as shown in Figure 1a. The curing time was set to 2 min, 4 min, 6 min, 8 min, and 10 min, five crosslinked EVA fibers were obtained and named as EVA-2M, EVA-4M, EVA-6M, EVA-8M, and EVA-10M, respectively.

### 2.3. Morphology Characterization

The EVA fiber was sputter-coated with gold and observed through scanning electron microscopy (FE-SEM) at an accelerated voltage of 3.0 kV. The diameter of samples was measured using a polarized light microscope (DM2700P, Leica, Wetzlar, Germany). The measurement of diameter distribution and coefficient of variation (*CV*) referred to the standard SN/T 2672-2010. The interval of EVA fiber diameter measurement was 5 mm with a specimen number of 300. The samples were placed in a chamber with constant temperature and humidity for 24 h (temperature of 20 ± 2 °C, relative humidity of 65% ± 4%) before testing.

### 2.4. Gel Fraction Measurement

Each crosslinked EVA fiber was wrapped with a 100 M copper mesh and immersed in xylene, and then, it was heated to 140 °C and kept for 12 h. The sample after extraction was completely dried in a vacuum oven at 60 °C. The gel mass fraction (*G*) of crosslinked EVA fiber was calculated by Equation (1):

(1) $$G=\frac{m_{2}-m_{0}}{m_{1}-m_{0}}\times 100\%$$

where *m*_0_ is the mass of the copper mesh, *m*_1_ and *m*_2_ are the mass of the total mass of sample and copper mesh before and after extraction, respectively.

### 2.5. Thermal and Mechanical Properties Tests

The crystallization and melting behavior of EVA fiber were characterized by differential scanning calorimetry (DSC Q2000, TA Instruments, New Castle, DE, America) under nitrogen. The fibers were first raised to 130 °C and insulation for 3 min to eliminate thermal history. Then, the samples were cooled to 20 °C and heated to 130 °C again. The heating change rate was 10 °C/min. The measurement of fractional crystallinity (*X*_c_) of samples referred to [50].

The room-temperature tensile tests of EVA fiber were conducted using a multi-purpose tensile tester (KES-G1, Kato Tech Co., Kyoto, Japan). Cylinder-shaped specimens with a length of 15 mm were stretched at a strain speed of 6 mm/min.

### 2.6. Shape Memory Experiments

1W- and 2W-SME of EVA fiber were measured by the TMA Q400 (TA Instruments, New Castle, DE, America) in dynamic DMA mode following previous research [11,42]. 1W-SME was investigated by the following four steps. First, the EVA fiber was raised to high temperature (*T*_high_ at 100 °C) and creep at 100 °C for 10 min. Second, the stress was added to obtain tensile strain. After stress was maintained for 5 min, the temperature was cooled to low temperature (*T*_low_ at 20 °C) and the stress was released. Finally, the temperature was reheated to *T*_high_ and the temporary shape of fiber recovered. The difference between 1W- and 2W-SME was that the stress applied in the second step was retained. The temperature and stress change rates were 10 °C/min and 0.05 MPa/min, respectively. The shape memory cycles under different stresses were repeated five times. Notably, the additional stress (ca. 0.17 MPa) was generated by the TMA fixture due to its own weight. Therefore, the sample was tested under the gravity of the TMA fixture. In the thermo-mechanical cycle curve, the thermal shrinkage strain fixation rate (*R*_s_) was determined by Equation (2):

(2) $$R_{s}=\frac{S_{s}-S_{c}}{S_{s}}\times 100\%$$

where *S*_s_ and *S*_c_ are the thermal shrinkage strain and creep strain of the fibers under 0.17 MPa.

The shape fixity ratio (*R*_f, 1W_) and shape recovery ratio (*R*_r,1W_) of 1W-SME and the actuation strain (*R*_a,2W_) and two-way shape recovery ratio (*R*_r,2W_) of 2W-SME were calculated by Equations (3)–(6) [42]:

(3) $$R_{f,1W}=\frac{\varepsilon_{unload}-\varepsilon_{inital}}{\varepsilon_{load}-\varepsilon_{inital}}\times 100\%$$

(4) $$R_{r,1W}=\frac{\varepsilon_{unload}-\varepsilon_{rec}}{\varepsilon_{unload}-\varepsilon_{inital}}\times 100\%$$

(5) $$R_{a,2W}=(\varepsilon_{low}-\varepsilon_{high})\times 100\%$$

(6) $$R_{r,2W}=\frac{\varepsilon_{low}-\varepsilon_{high}}{\varepsilon_{low}-\varepsilon_{0}}\times 100\%$$

In Equations (3) and (4), *Ɛ*_inital_ is the strain of the sample at *T*_high_ before applying stress, *Ɛ*_load_ is the maximum strain under load, *Ɛ*_unload_ is the fixed strain after cooling and stress removal, and *Ɛ*_rec_ is the strain after reheating and recovery. In Equations (5) and (6), *Ɛ*_0_ represents the strain after programming at *T*_high_ and *Ɛ*_low_ correspond to the strain after a complete crystallization-induced elongation at *T*_low_, respectively. Moreover, *Ɛ*_high_ represents the strain after complete melting-induced contraction caused by reheating to *T*_high_.

## 3. Results and Discussion

### 3.1. Morphology of EVA Fiber

**Figure 2:**
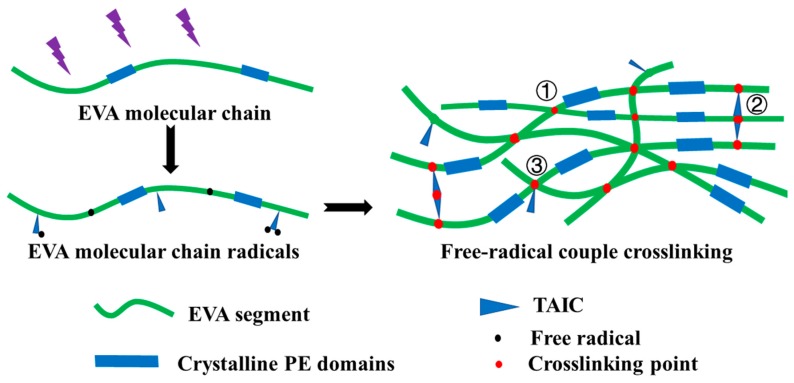
Schematic illustration of the crosslinking EVA fiber via free radical reaction initiated by UV.

Melted EVA mixed with BP and TAIC was extruded and drawn using laboratory-made drawing equipment. EVA continuous filament was stably prepared by efficient and convenient melt spinning (see Figure 1b), and the average diameter of the EVA fiber was 261.86 ± 13.07 μm (*CV* = 4.99%) as shown in Figure 1e,f. In the second step, EVA fiber with chemical crosslinking network was fabricated by UV curing, and BP and TAIC were photoinitiator and multi-functional crosslinking agent, respectively. The gel fraction (G) of EVA fibers was in the range of 1.62–43.68% according to the different UV curing time, indicated that the crosslink density of EVA fiber could be conveniently adjusted by changing the UV curing time [51]. When exposed to UV lamp, the initiator benzophenone changes from the ground state to the excited state after absorbing UV energy, and the excited benzophenone can undergo hydrogen abstraction reaction with the EVA and TAIC molecular chains, leading to the generation free radicals. Therefore, the free radicals of the EVA molecular chain can not only crosslink with other EVA molecular chains (reaction 1) but also react with the TAIC molecular groups (reaction 2 and 3), as shown in Figure 2. The addition of TAIC helps to reduce chain scission reactions and increase crosslinking of EVA fiber [52,53]. UV curing at room temperature preserves the complete structure of EVA fiber, and the morphology after crosslinking was shown in Figure 1c,d. The regular cylindrical profile and smooth surface of the fiber were apparent. Owing to the mild conditions of UV curing, no significant change in fiber structure was observed. This method is considered efficient and convenient for the manufacture of crosslinking EVA fiber.

### 3.2. Thermal and Mechanical Properties of EVA Fiber

**Figure 3:**
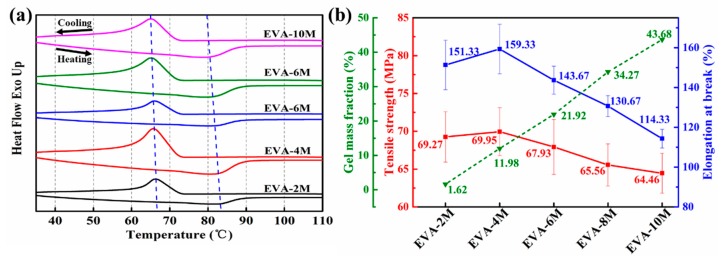
(**a**) DSC heating and cooling curves and (**b**) physical properties of different EVA fiber.

The values of *T*_m_ and *T*_c_ were calculated as the peak temperature of the corresponding heat flow curves as shown in Figure 3 and listed in Table 1. *T*_m_ slightly decreased from 82.37 to 79.63 °C (the standard deviation of *T*_m_ was 0.953) with the curing time increasing. The same decreasing trend was obtained for *T*_c_ and *X*_c_ (the standard deviation of *T*_c_ and *X*_c_ was 0.371 and 0.994, respectively). This observation was caused by the increase in crosslink density, which impedes the movement of the molecular chain and crystal growth. Meanwhile, the self-adjustment ability and chain mobility of the chain segments decreased due to the crosslinked network and then restrict the growth of EVA crystals [40,44]. Owing to good orientation in the drafting process, the mechanical properties of EVA fiber have been remarkably enhanced. When the curing time was 4 min, the tensile strength and elongation at break reached a maximum of 69.95 MPa and 159.33%, respectively. The mechanical properties can be improved by suitable crosslinking. On the contrary, the tensile strength lightly decreased from 67.93 MPa to 64.46 MPa when the curing time increased from 6 min to 10 min. A similar trend was noticed for elongation at break between 143.66% and 114.33% (see Table 1). This result may be due to UV aging [54]. It is worth noting that the mechanical properties of EVA fiber was improved by nearly 485% compared to the reported two-way shape memory EVA film, owing to the molecular chain orientation which was realized in melting spinning [42].

### 3.3. Creep and 2W-SME of EVA Fiber

**Figure 4:**
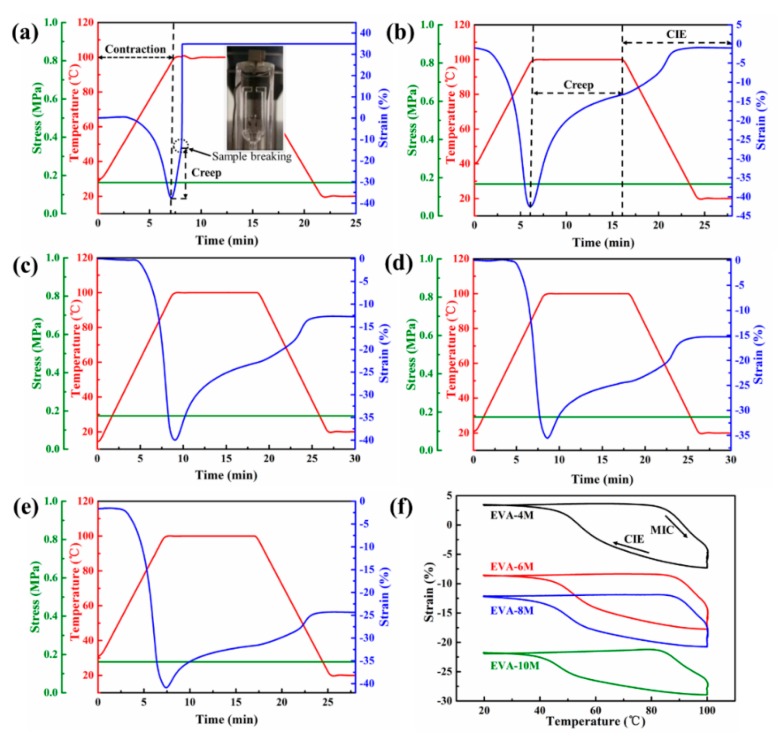
Thermal contraction and creep behavior under a constant stress of 0.17 MPa for (**a**) EVA-2M, (**b**) EVA-4M, (**c**) EVA-6M, (**d**) EVA-8M, and (**e**) EVA-10M. (**f**) Two-way shape memory effect (2W-SME) cycle curves of EVA fiber under a constant stress of 0.17 MPa with different UV curing time.

The thermal shrinkage and creep behavior of EVA fiber with different curing time were shown in Figure 4. All samples exhibited approximately 40% heat shrinkage during the heating process. Notably, the crosslink density of EVA-2M was too low to break, which evidently did not achieve shape memory (Figure 4a). The different creep behaviors were observed in Figure 4b–e, the *R*_s_ increased from 29.14% to 76.39% as the curing time increased. Therefore, the reversible shape memory transition in different strain ranges was exhibited under 0.17 MPa. The plastic flow of the molecular chains was limited by increasing the crosslink density of EVA fiber, which increases its cyclic stability. Moreover, the significant elongation occurred during the cooling process (see Figure 4b–e). The 2W-SME cycles of EVA-4M, -6M, -8M, and -10M under 0.17 MPa in detail as presented in Figure 4f. EVA-4M had a large reversible strain (>10%) under 0.17 MPa, but its cycle stability was poor due to large creep. In fact, SME strongly depends on the crosslink density and deformation stress [36,40]. Therefore, EVA-6M, EVA-8M, and EVA-10M were chosen as the study components and discussed the effects of different crosslink densities and deformation stresses on 1W-SME and two-way reversible strain comprehensively.

### 3.4. 1W- and 2W-SME of EVA Fiber

**Figure 5:**
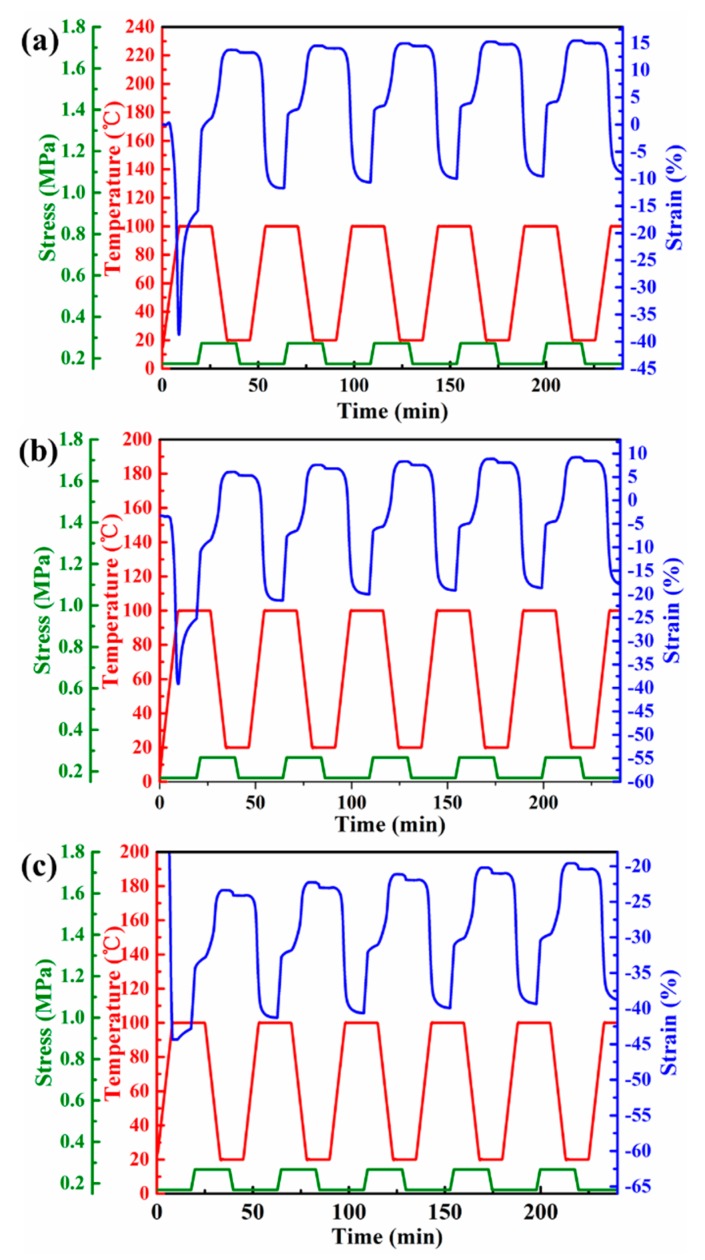
1W-SME cycles under a constant stress of 0.27 MPa for (**a**) EVA-6M, (**b**) EVA-8M, and (**c**) EVA-10M.

**Figure 6:**
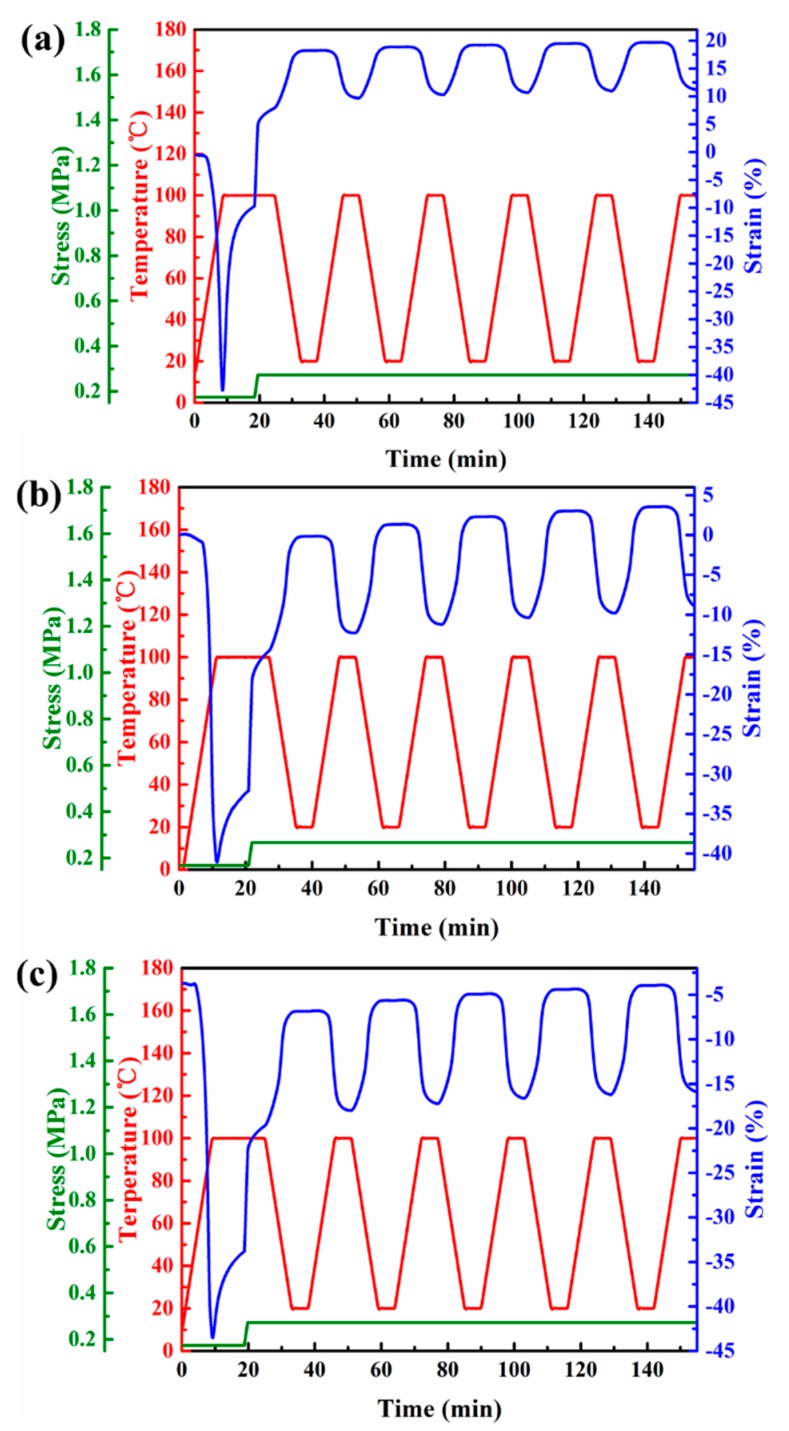
2W-SME cycles under a constant stress of 0.27 MPa for (**a**) EVA-6M, (**b**) EVA-8M, and (**c**) EVA-10M.

EVA fibers with large crosslink density were chosen to test 1W- and 2W-SME for avoiding breakage of the samples. The fiber underwent heating-cooling cycles between 20 and 100 °C five times under 0.27 MPa (Figure 5 and Figure 6). The *R*_f,1W_ and *R*_r,1W_ of different EVA fiber for each heating-cooling cycle were calculated from Figure 5. They remain nearly constant (all higher than 95%) as the crosslink density increased (Table 2). This result is consistent with previous research by Li et al. [36]. However, it should be noted that shape memory transforms in different strain ranges was observed due to the heat shrinkage and creep. The plastic flow of the molecular chain was limited due to the increased crosslink density of EVA fibers, resulting in the improved thermal mechanical property. Therefore, the shape memory transition in different strain ranges was exhibited when the external stress was increased to 0.27 MPa. For example, Figure 5a,c show the shape memory process in the stretched and contracted states (relative to the initial length), respectively. Moreover, the significant MIC and CIE emerge during each heating-cooling cycle [55]. The *R*_a,2W_ of EVA-6M was only 8.69% after five cycles, and there was a downward trend as shown in Figure 5a and Table 3. Most importantly, the *R*_a,2W_ of EVA-8M and EVA-10M was 13.35% and 12.26%, respectively. It was larger than EVA-6M under the same crystallization condition and stress. Previous research had shown that the constrained effect of the crosslinked network influences the oriented crystallization under different stresses [56,57]. EVA-6M with a relatively low crosslink density does not achieve the expected two-way shape memory effect despite its good one-way shape performance. Overall, Figure 5 and Figure 6 showed that: An appropriate crosslink density was indispensable to ensure larger loads and greater reversible strain.

### 3.5. Effect of Crosslink Density and Applied Stress on Reversible Strain of EVA Fiber

**Figure 7:**
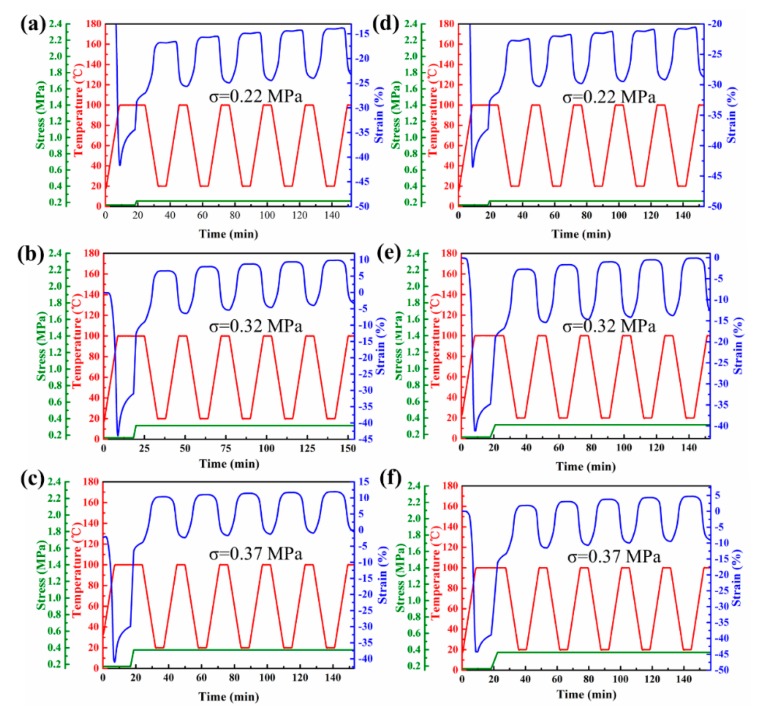
2W-SME cycles of (**a**–**c**) EVA-8M, and (**d**–**f**) EVA-10M with increasing constant stress.

**Figure 8:**
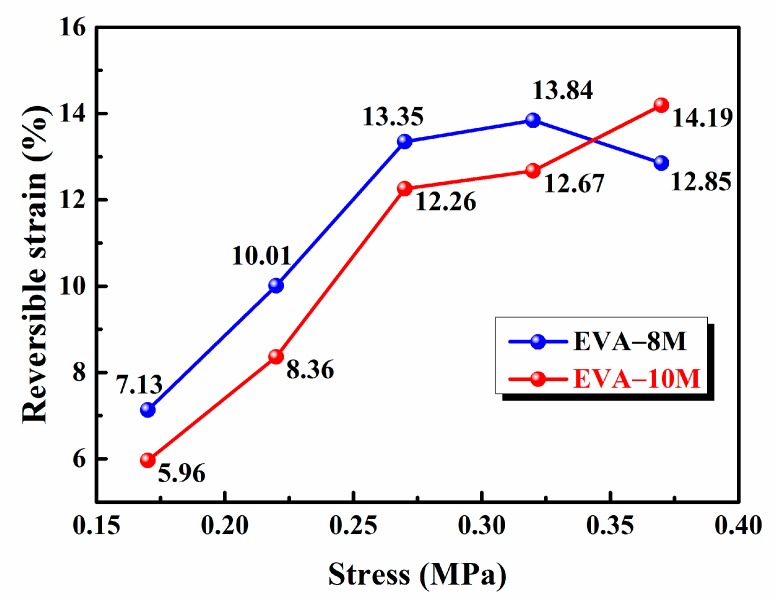
Effect of UV curing time and applied stress on the reversible strain of the crosslinked EVA fibers. (Reversible strain was calculated by the fifth heating-cooling cycle).

EVA-8M and EVA-10M were chosen to evaluate the reversible strain of EVA fiber with different crosslink densities. EVA-8M was subjected to different constant stress (0.22 MPa, 0.32 MPa, and 0.37MPa) and the results from five consecutive cycles were illustrated in Figure 7a–c. The same shape memory experiment profile was applied to EVA-10M (Figure 7d–f). The relationship between applied stress and reversible strain was illustrated in Figure 8. The *R*_a,2W_ of EVA-8M increased with applied stress increase and reached a maximum value of 13.84% when the stress increased to 0.32 MPa (see Figure 8). The degree of molecular chain orientation of EVA fiber was increased by increased external stress, which provides an additional template skeleton for the recrystallization of the segments, further increasing the reversible strain during cooling [42,53]. When the stress was further increased to 0.37 MPa, the *R*_a,2W_ of EVA-10M increased to 14.19%, *R*_a,2W_ of EVA-8M reduced to 12.83%. This phenomenon was attributed to the competitive relationship between the crosslinked network and the crystalline phase [58,59]. The crosslinked network memorized the initial shape and provides entropy elasticity for shape recovery. The entropic elastic restoring force of EVA fiber increased with the crosslink density increasing. When the external stress increased continuously, the segmental motion of EVA-8M was limited, and further, its recrystallization ability was limited. Therefore, the reversible strain value of EVA-10M with a large crosslink density is higher than that of EVA-8M.

### 3.6. Macroscopic Thermo-Actuated 2W-SME of EVA Fiber under Constant Stress

**Figure 9:**
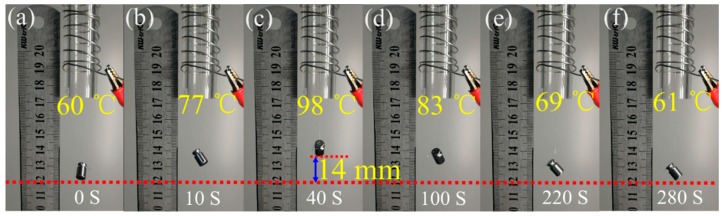
Digital photographs showing the 2W-SME of the EVA-10M fiber under constant stress.

**Figure 10:**
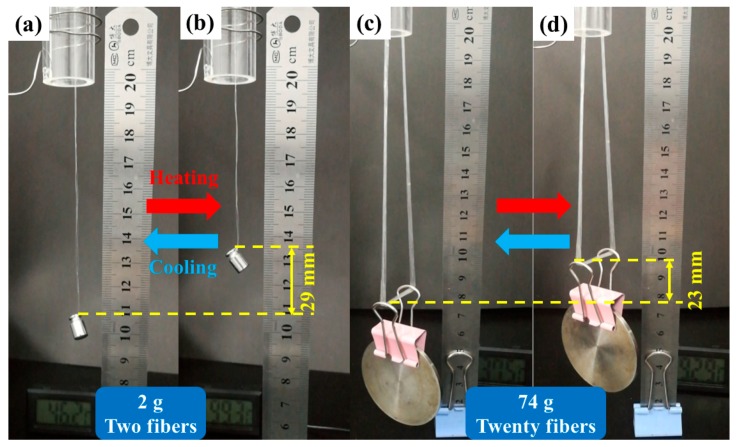
The reversible actuation capability was increased by combing multiple fibers.

The macroscopic reversible shape memory behavior by using electric heating quartz tube as shown in Figure 9. The length of the heating tube was 15 cm. EVA-10M fiber passed through the quartz heating tube and hung a 2 g weight. The heating of the quartz tube was achieved using a fixed resistance wire external to the tube. The macroscopic thermo-actuated 2W-SME was observed. The weight increased rapidly (approximately 14 mm) within 40 s during the heating process as illustrated in Figure 9a–c and it dropped slowly during the cooling process (see Figure 9d–f). The single fiber could lift more than 143 times its own weight and had a 9% reversible actuation strain. One of the important characteristics of fibers is the flexibility in structural design. A 2 g weight was hung on one end of a folded single fiber, as shown in Figure 10a,b. Upon heating, EVA fiber contracted and lifted the 2 g weight with a stroke of 29 mm, showed a reversible strain of 19%. A much higher driving force can be applied after winding a single fiber into a coil, as shown in Figure 10c,d. The number of load-bearing fibers in the fiber bundle has increased to 20 and was able to lift an object of 74 g with a comparable reversible displacement. It indicated that the applicability of this 2W-SME EVA fiber could be readily realized through simple structural design, which was restricted in other bulky 2W-SME systems. The reversible shape memory capability of EVA fiber makes them excellent candidate materials for smart textiles, flexible sensor, and actuator applications.

## 4. Conclusions

The 2W-SME of EVA fiber was successfully fabricated by melt spinning and UV curing. The properties of two-way shape memory EVA fiber can be easily adjusted by simply varying the UV curing time. TMA thermo-mechanical test revealed that EVA fiber with the suitable crosslink networks exhibited excellent 1W-SME with *R*_f, 1W_ and *R*_r, 1W_ of more than 95%. Moreover, the CIE phenomenon appeared in 1W-SME cycle, which was crucial for achieving 2W-SME. Systematic evaluation indicated that the crosslink density and applied stress greatly influenced the 2W-SME. EVA-10M with higher gel fraction could withstand high loads (stress of 0.37 MPa) and had a reversible strain of 14.19%. Macroscopic thermo-actuated 2W-SME showed that EVA-10M fiber could lift more than 143 times its own weight and achieve 9% reversible actuation. The reversible actuation capability was significantly enhanced by a simple winding design of the single EVA fiber. The design of two-way shape memory EVA fiber has the advantages of low equipment requirements, low cost, and convenient operation. It is expected to realize the application of commercial polymers in the fields of smart textiles, flexible actuators, and artificial muscles.

## Figures and Tables

**Table 1:** Physical and mechanical properties of different EVA fiber.

Samples	*T*_m_ (°C)	*T*_c_ (°C)	*G* (%)	*X*_c_ (%)	Tensile Strength (MPa)	Elongation at Break (%)
EVA-2M	82.37	66.25	1.62	19.42	69.27 ± 3.34	151.33 ± 12.39
EVA-4M	81.87	65.82	11.98	18.73	69.95 ± 3.16	159.33 ± 12.40
EVA-6M	81.25	65.75	21.92	17.60	67.93 ± 3.63	143.66 ± 7.07
EVA-8M	80.69	65.16	34.27	16.86	65.56 ± 2.78	130.67 ± 5.27
EVA-10M	79.63	65.42	43.68	17.02	64.46 ± 2.65	114.33 ± 4.65

**Table 2:** One-way (1W)-SME of different EVA fiber under 0.27 MPa.

Cycles	EVA-6M		EVA-8M		EVA-10M	
	*R*_f,1W_ (%)	*R*_r,1W_ (%)	*R*_f,1W_ (%)	*R*_r,1W_ (%)	*R*_f,1W_ (%)	*R*_r,1W_ (%)
1	98.39	85.32	97.58	87.88	95.86	92.28
2	98.21	95.73	97.33	95.20	95.94	96.32
3	98.24	97.25	97.13	98.52	95.73	96.19
4	98.06	98.14	97.18	98.09	95.83	96.82
5	98.01	98.86	97.16	98.19	95.13	97.51

**Table 3:** 2W-SME of different EVA fiber under 0.27 MPa.

Cycles	EVA-6M		EVA-8M		EVA-10M	
	*R*_a,2W_ (%)	*R*_r,2W_ (%)	*R*_a,2W_ (%)	*R*_r,2W_ (%)	*R*_a,2W_ (%)	*R*_r,2W_ (%)
1	10.32	84.24	13.75	86.88	12.20	93.59
2	9.18	93.68	13.62	92.07	12.33	93.59
3	8.94	95.53	13.50	93.56	12.26	95.27
4	8.81	96.48	13.35	96.03	12.20	96.72
5	8.69	97.12	13.35	96.23	12.26	97.39
